# Integration of hand hygiene in health-care workers behavior

**DOI:** 10.1186/1753-6561-5-S6-P106

**Published:** 2011-06-29

**Authors:** N Khamis

**Affiliations:** 1 Infection Control, Ain Shams University Specialized Hospital, Cairo, Egypt

## Introduction / objectives

Healthcare workers (HCWs) hands play a crucial role in the transmission of micro organisms. Therefore, hand hygiene is acknowledged as the single most effective measure to prevent healthcare-associated infection. However many factors are interplaying making this simple, most effective preventive action not easy to be achieved, some are related to tools and others are in direct relation to behavioral incompetence of HCWs.

## Methods

In a 1000 beds university hospital in Cairo, an infection control system was founded since 1989, and hand hygiene is one of the policies implemented in all hospital departments. Since 2008, system change, from hand washing to alcohol-based hand rub, was followed. Tools were provided in the form of WHO guidelines, educational material, and supplies as alcohol-based products (dispensers and pocket bottles). On-job training and follow-up was conducted to embed the new concept of hand hygiene and to measure the compliance among HCWs (nurses and doctors).

Two hand hygiene campaigns were conducted over 2009 and 2010. The former was for two weeks and assessment using a 5-moments designed observation sheet was used to measure the compliance. The later was for one week using the first moment observation sheet of WHO.

## Results

For 2009, compliance was manually calculated in six departments and it ranged from 10.8% to 89% for **moment one**, 4% to 100% for **moment two**, 86% to 100% for **moment three**, 70.9% to 100% for **moment four** and for **moment five** 61% to 100%. In 2010, automatique calculation of compliance for moment one was 48.91% in five medical departments.

## Conclusion

Integration of hand hygiene in HCWs behavior needs a long persistent educational program as well as continuous assessment of level of knowledge. Regular awareness courses for both nurses and doctors and provision of tools as alcohol-based products are recomended, to help for proper behavior change.

## Disclosure of interest

None declared.

